# Microwave-Assisted Condensation Reactions of Acetophenone Derivatives and Activated Methylene Compounds with Aldehydes Catalyzed by Boric Acid under Solvent-Free Conditions

**DOI:** 10.3390/molecules200611617

**Published:** 2015-06-23

**Authors:** Elodie Brun, Abdelmounaim Safer, François Carreaux, Khadidja Bourahla, Jean-Martial L’helgoua’ch, Jean-Pierre Bazureau, Jose Manuel Villalgordo

**Affiliations:** 1 Institut des Sciences Chimiques de Rennes, Université de Rennes 1, Campus de Beaulieu, UMR 6226 CNRS, 35042 Rennes Cedex, France; E-Mails: brun_elodie@hotmail.com (E.B.); bkhadije@yahoo.fr (K.B.); jm.lhelgoualch@gmail.com (J.-M.L.); jean-pierre.bazureau@univ-rennes1.fr (J.-P.B.); 2 LSOA—Laboratoire de Synthèse Organique Appliquée, Department of chemistry, Oran 1 University, 1524 Oran, Algeria; E-Mail: asafer2001@yahoo.fr; 3 Villapharma Research, Parque Tecnologico de Fuente Alamo, E-30320 Murcia, Spain; E-Mail: jmvillalgordo@villapharma.com

**Keywords:** condensation reaction, boric acid, catalysis, solvent-free, microwave

## Abstract

We here disclosed a new protocol for the condensation of acetophenone derivatives and active methylene compounds with aldehydes in the presence of boric acid under microwave conditions. Implementation of the reaction is simple, healthy and environmentally friendly owing to the use of a non-toxic catalyst coupled to a solvent-free procedure. A large variety of known or novel compounds have thus been prepared, including with substrates bearing acid or base-sensitive functional groups.

## 1. Introduction

The development of new methodologies employing oxygenated boron compounds (*i.e.*, borinic acids R^1^R^2^B(OH), boronic acids RB(OH)_2_ and boric acid H_3_BO_3_) as reaction catalysts has received a lot of attention from the organic chemistry community during these past few years [1]. Beside their stability to air and moisture, the growing interest for this class of compounds can also be attributed to the different modes of catalytic reactivity achieved depending on their electronic nature. They can indeed act as Lewis acids but also as activators of functional groups such as hydroxyl and carboxylic groups by reversible covalent interactions [2]. Among them, boric acid was found to be an efficient catalyst in numerous reactions such as selective esterification of α-hydroxycarboxylic and malonic acids [3,4,5], amide formation from carboxylic acids [6,7,8], transamidation of carboxamides [9], trimethylsilylation of alcohols and phenols [10], decarboxylation of cyclic β-enaminoketoesters [11], ipso-hydroxylation of aryl boronic acids [12], aza [13] and thia-Michael addition [14], Friedel-Crafts alkylation of indoles [15] as well as in diverse multicomponent reactions [16,17,18,19].

**Figure 1:**
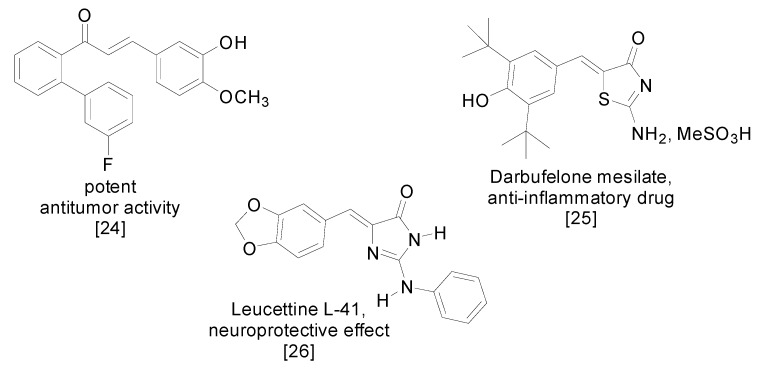
Selected molecules with high biological value.

Condensation reactions of aldehydes with active methylene compounds are very useful to prepare molecules with potential therapeutic relevance such as, for instance, the 1,3-diphenylpropenones also called chalcones. They exhibit diverse pharmacological activities [20,21,22] and more particularly, as anticancer agents [23,24]. Many other drugs as well as pharmacological tools with heterocyclic structures can also include an aldol-type condensation step in their synthesis [25,26], as illustrated in Figure 1.

These condensation reactions can be promoted by a large array of catalysts including Lewis bases and acids but can suffer from limitations related, among other factors, to waste elimination and use of toxic products [27]. Therefore, the development of new methods involving the environmentally benign and cost-efficient catalysts still remains a major challenge. In this context, the use of boric acid as a catalyst in these reactions should be particularly adapted due to its lack of apparent toxicity and its easy removal from a reaction mixture [28]. To the best of our knowledge, only a limited number of articles report the use of boric acid acting as the sole catalyst for aldol condensation and subsequent dehydration to form α,β-unsaturated ketones [29,30]. However, the described conditions require long reaction times at reflux of *m*-xylene in presence of 50 mol % of catalyst and water removal through a Dean-Stark trap.

As part of our program focused on the development of novel green methods based on organoboron chemistry for the synthesis of molecules with a high biological profile [31,32,33,34,35], we here report our efforts to develop a general procedure for condensation reactions employing boric acid as catalyst. The application of microwave technology as non-conventional energy source was crucial for the implementation of this methodology. Under solvent-free conditions, we have shown that the condensation protocol was efficient from various substrates bearing an activated methylene group. In addition, we found that the condensation reaction with an aldehyde possessing a boronic acid group proceeds without the need to an additional catalyst.

## 2. Results and Discussion

For the experimental protocol development, we selected the 4-methoxyacetophenone **1a** (1 equiv.) and the 4-methoxybenzaldehyde (1.2 equiv.) as model substrates due to the fact that, from a biological point of view, the chalcones bearing electron rich groups are part of the most interesting molecules [20]. After observing very low formation of the expected product **2a**, under thermal heating in presence of boric acid for 4 h (Table 1, entry 1), we tried different conditions using microwave irradiation as energy source and without adding any solvent. For a first attempt, we employed 50 mol % of boric acid at 160 °C for 20 min. Under these conditions, chalcone **2a** is formed stereoselectively (only *E*) with an almost complete conversion (95%) compared to 4-methoxyacetophenone (entry 2). Improvement of the reaction conversion when phenyl boronic acid is used instead of boric acid confirms that the catalyst has to act as a Lewis acid during the condensation reaction (entry 3).

**Table 1:** Optimization of the condensation reaction catalyzed by boric acid ^a^. 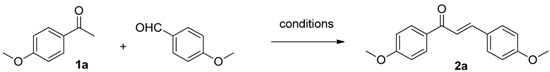

Entry	Catalyst (mol %)	Conditions	2a (Conv. %) ^c^	2a (yield %) ^d^
1	B(OH)_3_ (50 mol %)	Toluene, reflux (4 h)	5%	-
2	B(OH)_3_ (50 mol %)	MWI, 160 °C (20 min) ^b^	95%	-
3	PhB(OH)_2_ (50 mol %)	MWI, 160 °C (20 min) ^b^	100%	-
4	B(OH)_3_ (10 mol %)	MWI, 160 °C (40 min) ^b^	75%	-
5	B(OH)_3_ (20 mol %)	MWI, 160 °C (40 min) ^b^	93%	70%

^a^ Reaction condition: 4-methoxyacetophenone (1 mmol), 4-methoxybenzaldehyde (1.2 mmol);^b^ Microwave irradiation of the reaction mixture was carried out in a glass tube sealed with a snap cap using the Explorer^®^24 CEM apparatus (CEM µ Waves, Saclay, France) (*P* = 300 W); ^c^ Conversion based on 4-methoxyacetophenone **1a** as the limiting reagent; ^d^ Isolated pure product.

Even though phenyl boronic acid is a stronger Lewis acid than boric acid and is easy to handle, we continued our study using boric acid because of its low cost and the fact that it is a more environmentally friendly reagent [36]. Eventually, by changing the irradiation time, we found that it was possible to reduce the quantity of catalyst down to 20 mol % while keeping a good conversion (entry 5). The desired compound **2a** has been obtained in a pure form in an acceptable yield (70%) by mere addition, after heating, of a mixture of solvent (H_2_O/EtOH) and filtration.

**Scheme 1:**
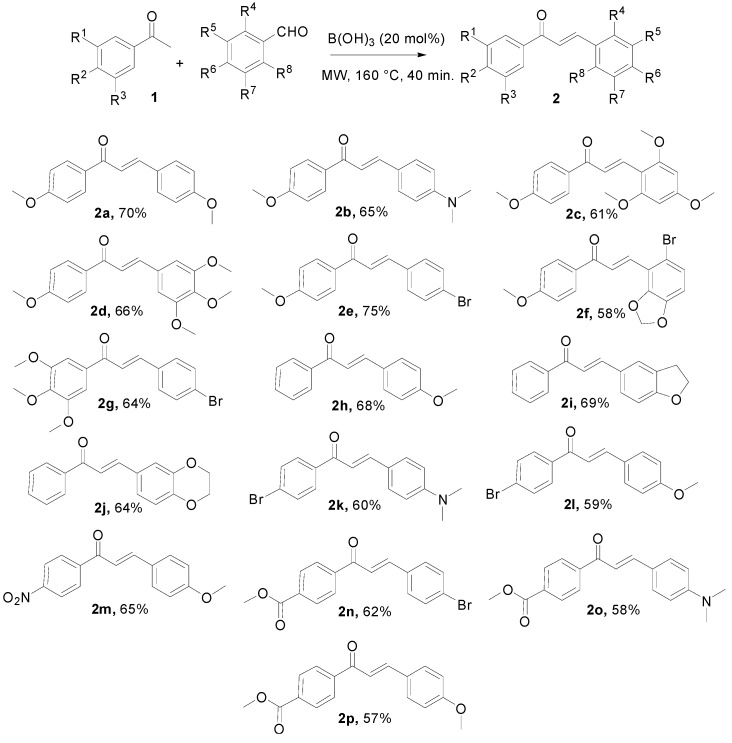
Synthesis of various functionalized chalcones.

The next goal was to extend this protocol to other acetophenone derivatives **1** and aldehydes in order to show the scope and limitations of this process. In all cases described in Scheme 1, chalcones **2** were obtained in a stereoselective manner (>95%) in favor of *E* isomer as confirmed by the magnitude of the coupling constant between the two vinyl protons (range from 15.5 to 15.8 Hz). The obtained yields after precipitation and filtration were not optimized but we observed slightly better results when bromo group was present on the aromatic ring of aldehydes (**2e** compared to **2a**, **2n** compared to **2o**). Undoubtedly, the solvent-free conditions are relatively mild compared to basic or acidic media generally implemented in conventional methods to prepare the 1,3-diaryl-2-propen-1-ones. As demonstrated by the synthesis of unknown compounds **2n**–**p**, this new process tolerates the presence of an ester functional group and allows access to functionalized chalcones which may be difficult to prepare by traditional methods. Typically, the aldol condensations in basic media are carried out in the appropriate alcoholic solvent to prevent the transesterification side-reaction. In this regard, our metal-free process can be considered as a complementary methodology of the catalyzed coupling reactions requiring any base such as the oxidative carbonylative vinylation reactions of aryl boronic acids with styrenes which is described in particular as compatible with the carbonyl groups [37].

**Scheme 2:**
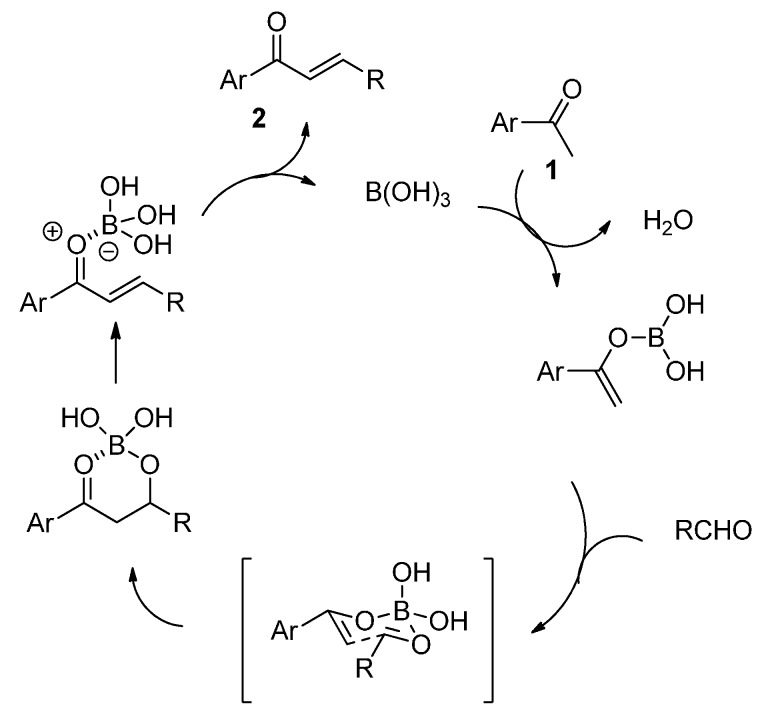
Proposed catalytic cycle for the formation of α,β-unsatured ketones.

Despite the lack of evidence for the postulated mechanism, we can speculate that, under these microwave irradiation conditions, the ketone first reacts with boric acid to form a boron enolate (Scheme 2). The aldol reaction between this species and an aldehyde might then proceed via a six-membered chair-like transition state. Finally, the resulting adduct may undergo a dehydration step assisted by a possible intramolecular coordination of the boron atom with the carbonyl group.

**Scheme 3:**
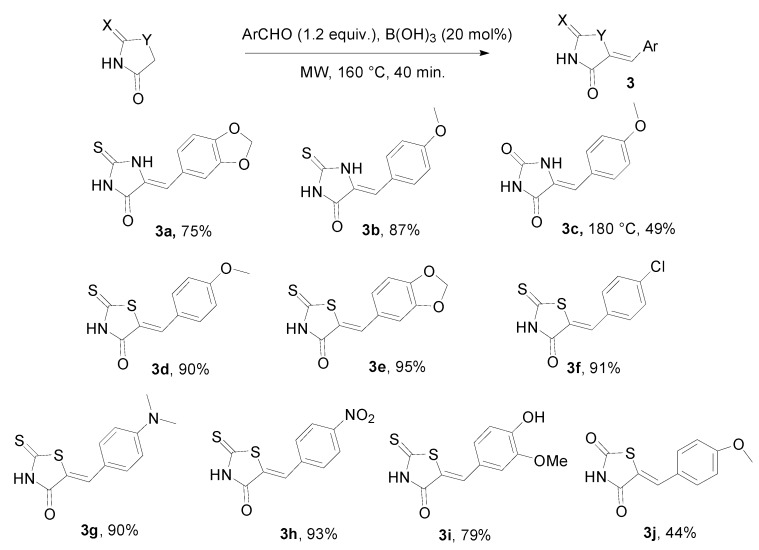
Condensation reaction with five-membered heterocyclic rings.

Five-membered heterocyclic rings such as the thiohydantoin (X = S, Y = NH), hydantoin (X = O, Y = NH), rhodanine or 2-thioxo-1,3-thiazolidin-4-one (X = S, Y = S) and 1,3-thiazolidin-2,4-dione (X = O, Y = S) have attracted our attention as potential substrates in our condensation protocol since they appear as interesting scaffolds for drug discovery [38]. To our delight, in all cases the expected products **3** were obtained in moderate to good yields (Scheme 3).

After microwave heating in the presence of 1.2 equivalent of aldehyde, the addition of ethanol in the crude reaction mixture allowed the formation of a precipitate that is filtered to give the 5-arylidene derivatives **3** as a sole isomer whose *Z* stereochemistry was assigned, in accordance with the literature. In the case of hydantoin, an adjustment of the experimental protocol was necessary to obtain compound **3c** in a suitable yield (49%). In a similar way, an increased temperature (180 °C) with thiazolidinedione as substrate was envisaged but without any substantial effect on yield (**3j**, 44%).

**Scheme 4:**
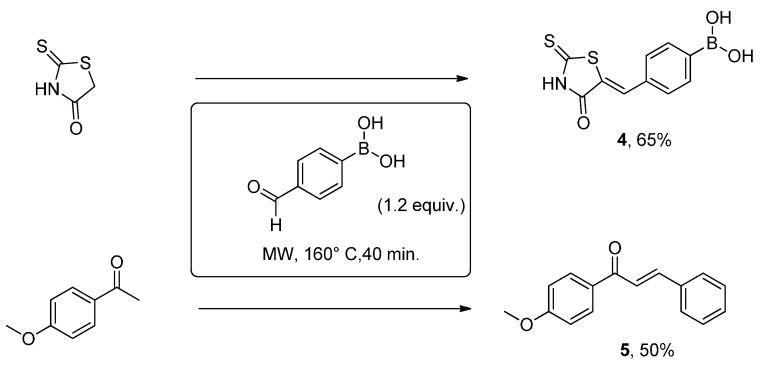
Condensation reactions involving the 4-formylboronic acid.

In these last two decades, boronic acid derivatives have become a significant class of compounds with important biological applications [39]. On this basis, we put forth the idea that the condensation reaction involving an aldehyde bearing a boronic group on the aromatic ring might constitute a new route to these organoboron compounds. Without the supplementary addition of catalyst, the reaction between thyohydantoin and 4-formylphenylboronic acid under microwave irradiation afforded the expected product **4** (Scheme 4).

The work-up is a simple filtration of the formed precipitate following the addition of a H_2_O/EtOH mixture in the crude mixture. Unfortunately and unexpectedly, when the reaction was achieved with 4-methoxyacetophenone using the same experimental conditions, product **5** rather than the chalcone containing the boronic acid function was isolated probably as a result of a protonolysis of the C-B bond. Other attempts were made with similar substrates though without any more success showing the difficulty to overcome this side reaction.

## 3. Experimental Section

All reagents were purchased from Acros (Geel, Belgium), Aldrich (Saint Louis, MI, USA) and were used without further purification. Melting points were determined on a Kofler melting point apparatus (Wagner & Munz, Munich, Germany), and were uncorrected. ^1^H-NMR spectra were recorded on BRUKER AC 300 P (300 MHz) spectrometer (Bruker, Bremen, Germany), ^13^C-NMR spectra on BRUKER AC 300 P (75 MHz, Bruker) spectrometer and ^11^B-NMR spectra on BRUKER AC 300 P (96 MHz, Bruker). Chemical shifts are expressed in part per million downfield from tetramethylsilane as an internal standard. Data are given in the following order: δ value, multiplicity (s, singlet; d, doublet; t, triplet; q, quartet; m, multiplet; br, broad), number of protons, coupling constants *J* is given in Hz. Reactions under microwave irradiations were realized in the Explorer^®^24 CEM microwave reactor (CEM France, CEM µ Waves) using borosilicate glass vials of 10 mL equipped with snap caps (at the end of the irradiation, cooling reaction was realized by compressed air). The microwave instrument consists of a continuous focused microwave power output from 0 to 300 W. All the experiments were performed using a stirring option. The target temperature was reached with a ramp of 2 min. and the chosen microwave power stayed constant to hold the mixture at this temperature. The reaction temperature is monitored using calibrated infrared sensor and the reaction time included the ramp period. The microwave irradiation parameters (power and temperature) were monitored by the ChemDriver software package (Version 2.0, CEM µ Waves). High-resolution mass sprectra (HMRS) were recorded on a Bruker Micro-Tof-Q II (Bruker) or on a Waters Q-Tof 2 at the CRMPO (Centre Régional de Mesures Physiques de l’Ouest, Rennes, France) using positive ion Electro-Spray Ionization (ESI, Waters, Manchester, UK). Purifications by column chromatography were all carried out on silica gel by using Acros silica 0.060–0.200 mm, 60 Å. Thin layer chromatography analyses were performed on Merck Silica Gel 60 F_254_ plates (Merck, Darmstadt, Germany).

### 3.1. General Procedure for the Condensation Reaction

A mixture of substrate (1 mmol), aldehyde (1.2 mmol) and boric acid (0.2 mmol) was placed in a cylindrical quartz reactor (Ø = 4 cm). The reactor was introduced into an Explorer^®^24 CEM apparatus. The stirred mixture was heated at 160 °C (*P* = 300 W) for 40 min, except for **3c** (180 °C). After microwave dielectric heating, the crude reaction mixture was allowed to cool down at room temperature and ethanol (10 mL) or mixture of H_2_O/EtOH (10 mL) was directly added in the cylindrical quartz reactor. The resulting precipitated product was filtered off and was purified by recrystallization from ethanol if necessary.

### 3.2. Physical and Spectroscopic Data of Products

*(E)-1*,*3-Bis-(4-methoxyphenyl)-prop-2-en-1-one* (**2a**) [40]. Pale cream powder, 70% yield, m.p. = 96–98 °C; ^1^H-NMR (CDCl_3_, 300 MHz): δ = 3.88 (s, 3H, OMe), 3.91 (s, 3H, OMe), 6.95 (d, 2H, *J* = 8.7 Hz, Ar-), 7.00 (d, 2H, *J* = 8.8 Hz, Ar-), 7.45 (d, 1H, *J* = 15.6 Hz, CH=), 7.62 (d, 2H, *J* = 8.7 Hz, Ar-), 7.80 (d, 1H, *J* = 15.6 Hz, CH=), 8.05 (d, 2H, *J* = 8.8 Hz, Ar-) ppm; ^13^C-NMR (CDCl_3_, 75 MHz): δ = 55.4, 55.5, 113.8, 114.4, 119.6, 127.8, 130.1, 130.7, 131.4, 143.8, 161.5, 163.3, 188.7 ppm; HRMS (ESI^+^): *m*/*z* calcd for C_17_H_16_O_3_ [M + Na]^+^ 291.0997; found 291.0996.

*(E)-3-(4-(Dimethylamino)phenyl)-1-(4-methoxyphenyl)-prop-2-en-1-one* (**2b**) [40]. light yellow powder, 65% yield, m.p. = 128–130 °C; ^1^H-NMR (CDCl_3_, 300 MHz): δ = 3.05 (s, 6H, N(CH_3_)_2_), 3.89 (s, 3H, OCH_3_), 6.71 (d, 2H, *J* = 8.7 Hz, Ar-), 6.99 (d, 2H, *J* = 8.4 Hz, Ar-), 7.37 (d, 1H, *J* = 15.4 Hz, CH=), 7.57 (d, 2H, *J* = 8.4 Hz, Ar-), 7.80 (d, 1H, *J* = 15.4 Hz, CH=), 8.01 (d, 2H, *J* = 8.7 Hz, Ar-) ppm; ^13^C-NMR (CDCl_3_, 75 MHz): δ = 40.2, 55.5, 111.8, 113.7, 116.6, 122.8, 130.3, 130.6, 131.9, 145.0, 151.9, 163.0, 188.9 ppm; HRMS (ESI^+^): *m*/*z* calcd for C_18_H_19_NO_2_ [M + Na]^+^ 304.1313; found 304.1313.

*(E)-1-(4-Methoxyphenyl)-3-(2*,*4*,*6-trimethoxyphenyl)-prop-2-en-1-one* (**2c**) [41]. Yellow powder, 61% yield, m.p. = 128–130 °C; ^1^H-NMR (CDCl_3_, 300 MHz): δ = 3.88 (s, 3H, OCH_3_), 3.90 (s, 3H, OCH_3_), 3.93 (s, 6H, OCH_3_), 6.16 (s, 2H, Ar-), 6.98 (d, 2H, *J* = 8.8 Hz, Ar-), 7.90 (d, 2H, *J* = 15.8 Hz, CH=), 8.04 (d, 2H, *J* = 8.8 Hz, Ar-), 8.24 (d, 1H, *J* = 15.8 Hz, CH=) ppm; ^13^C-NMR (CDCl_3_, 75 MHz): δ = 55.8, 56.5, 56.8, 57.3, 97.5, 112.1, 114.1, 116.2, 120.6, 131.1, 132.1, 139.7, 143.7, 152.7, 154.9, 163.5, 189.7 ppm; HRMS (ESI^+^): *m*/*z* calcd for C_19_H_20_O_5_ [M + Na]^+^ 351.1208; found 351.1208.

*(E)-1-(4-Methoxyphenyl)-3-(3*,*4*,*5-trimethoxyphenyl)-prop-2-en-1-one* (**2d**) [42]. Pale yellow powder, 66% yield, m.p. = 138–140 °C; ^1^H-NMR (CDCl_3_, 300 MHz): δ = 3.92 (s, 6H, OCH_3_), 3.95 (s, 6H, OCH_3_), 6.88 (s, 2H, Ar-), 7.01 (d, 2H, *J* = 8.7 Hz, Ar-), 7.43 (d, 1H, *J* = 15.8 Hz, CH=), 7.73 (d, 1H, *J* = 15.8 Hz, CH=), 8.05 (d, 2H, *J* = 8.7 Hz, Ar-) ppm; ^13^C-NMR (CDCl_3_, 75 MHz): δ = 55.9, 56.6, 61.4, 106.0, 114.2, 121.7, 131.0, 131.2, 131.5, 140.7, 144.5, 153.9, 163.8, 189.1 ppm; HRMS (ESI^+^): *m*/*z* calcd for C_19_H_20_O_5_ [M + Na]^+^ 351.1208; found 351.1209.

*(E)-3-(4-Bromophenyl)-1-(4-methoxyphenyl)prop-2-en-1-one* (**2e**) [43]. Pale yellow powder, 75% yield, m.p. = 157–159 °C; ^1^H-NMR (CDCl_3_, 300 MHz): δ = 3.92 (s, 3H, OCH_3_), 7.00 (d, 2H, *J* = 8.9 Hz, Ar-), 7.49–7.61 (m, 5H, Ar, CH=), 7.75 (d, 1H, *J* = 15.6 Hz, CH=), 8.05 (d, 2H, *J* = 8.9 Hz, Ar-) ppm; ^13^C-NMR (CDCl_3_, 75 MHz): δ = 55.5, 113.9, 122.4, 124.5, 129.7, 130.8, 130.9, 132.1, 134.0, 142.5, 163.5, 188.3 ppm; HRMS (ESI^+^): *m*/*z* calcd for C_16_H_13_BrO_2_ [M + Na]^+^ 338.9997; found 338.9996.

*(E)-3-(5-Bromobenzo[d][1,3]dioxol-4-yl)-1-(4-methoxyphenyl)prop-2-en-1-one* (**2f**). Pale green powder, 58% yield, m.p. = 120–122 °C; ^1^H-NMR (CDCl_3_, 300 MHz) δ= 3.89 (s, 3H, OCH_3_), 6.16 (s, 2H, OCH_2_O), 6.72 (d, 1H, *J* = 8.3 Hz, Ar-), 7.01 (d, 2H, *J* = 8.9 Hz, Ar-), 7.15 (d, 1H, *J* = 8.3 Hz, Ar-), 7.90 (d, 1H, *J* = 15.8 Hz, CH=), 8.01 (d, 1H, *J* = 15.8 Hz, CH=), 8.07 (d, 2H, *J* = 8.9 Hz, Ar-) ppm; ^13^C-NMR (CDCl_3_, 75 MHz): δ = 55.4, 102.1, 110.1, 113.8, 117.0, 118.2, 125.9, 127.2, 130.9, 137.4, 147.3, 147.5, 163.5, 188.8 ppm; HRMS (ESI^+^): *m*/*z* calcd for C_17_H_13_BrO_4_ [M + Na]^+^ 382.9895; found 382.9895.

*(E)-3-(4-Bromophenyl)-1-(3*,*4*,*5-trimethoxyphenyl)prop-2-en-1-one* (**2g**) [44]. Pale yellow powder, 64% yield, m.p. = 123–124 °C; ^1^H-NMR (CDCl_3_, 300 MHz): δ: 3.92 (s, 3H, OCH_3_), 3.93 (s, 6H, 2 × OCH_3_), 7.22 (s, 2H, Ar-), 7.42 (d, 1H, *J* = 15.6 Hz, CH=), 7.48 (d, 2H, *J* = 8.0 Hz, Ar-), 7.54 (d, 2H, *J* = 8.0 Hz, Ar-), 7.72 (d, 1H, *J* = 15.6 Hz, CH=) ppm; ^13^C-NMR (CDCl_3_, 75 MHz): δ = 56.6, 56.7, 61.2, 106.4, 122.5, 125.0, 130.0, 131.7, 132.5, 133.5, 134.0, 143.5, 153.4, 189.3 ppm; HRMS (ESI^+^): *m*/*z* calcd for C_18_H_17_BrO_4_ [M + Na]^+^ 399.0208; found 399.0208.

*(E)-3-(4-Methoxyphenyl)-1-phenylprop-2-en-1-one* (**2h**) [43]. Pale yellow powder, 68% yield, m.p. = 74–76 °C; ^1^H-NMR (CDCl_3_, 300 MHz): δ: 3.83 (s, 3H, OCH_3_), 6.96 (d, 2H, *J* = 8.8 Hz, Ar-), 7.44 (d, 1H, *J* = 15.7 Hz, CH=), 7.49–7.58 (m, 3H, Ar-), 7.63 (d, 2H, *J* = 8.8 Hz, Ar-), 7.80 (d, 1H, *J* = 15.7 Hz, CH=), 8.03 (d, 2H, *J* = 8.1 Hz, Ar-) ppm; ^13^C-NMR (CDCl_3_, 75 MHz): δ = 55.4, 119.8, 127.7, 128.5, 128.6, 130.2, 130.3, 132.6, 138.5, 144.7, 161.6, 190.6 ppm. HRMS (ESI^+^): *m*/*z* calcd for C_16_H_14_O_2_ [M + Na]^+^ 261.0891; found 261.0893.

*(E)-3-(2,3-Dihydrobenzofuran-5-yl)-1-phenylprop-2-en-1-one* (**2i**). Pale yellow powder, 69% yield, m.p. = 110–112 °C; ^1^H-NMR (CDCl_3_, 300 MHz): δ= 3.26 (t, 2H, *J* = 8.7 Hz, ArCH_2_), 4.65 (t, 2H, *J* = 8.7 Hz, CH_2_O), 6.82 (d, 1H, *J* = 8.3 Hz, Ar-), 7.31 (d, 1H, *J* = 15.6 Hz, CH=), 7.41–7.61 (m, 5H, Ar-), 7.79 (d, 1H, *J* = 15.6 Hz, Ar-), 8.01 (dd, 2H, *J* = 1.6, 8.5 Hz, Ar-) ppm; ^13^C-NMR (CDCl_3_, 75 MHz): δ= 29.1, 71.8, 109.7, 118.9, 124.8, 127.6, 128.1, 128.2, 128.4, 130.0, 132.4, 138.4, 145.1, 162.5, 190.4 ppm. HRMS (ESI^+^): *m*/*z* calcd for C_17_H_14_O_2_ [M + Na]^+^ 273.0891; found 273.0891.

*(E)-3-(2,3-Dihydrobenzo[b][1,4]dioxin-6-yl)-1-phenyl-prop-2-en-1-one* (**2j**) [45]. Yellow powder, 64% yield, m.p. = 82–84 °C; ^1^H-NMR (CDCl_3_, 300 MHz): δ = 4.30 (m, 4H, OCH_2_CH_2_O), 6.90 (d, 1H, *J* = 8.2 Hz, Ar-), 7.14–7.20 (m, 2H, Ar-), 7.38 (d, 1H, *J* = 15.6 Hz, CH=), 7.47–7.6 (m, 3H, Ar-), 7.71 (d, 1H, *J* = 15.6 Hz, CH=), 8.00 (dd, 2H, *J* = 1.6, 8.5 Hz, Ar-) ppm; ^13^C-NMR (CDCl_3_, 75 MHz): δ = 64.2, 64.6, 116.7, 117.0, 117.8, 120.4, 121.2, 122.6, 128.0, 128.4, 128.6, 132.6, 138.5, 143.8, 144.6, 146.0, 190.5 ppm; HRMS (ESI^+^): *m*/*z* calcd for C_17_H_14_O_3_ [M + Na]^+^ 289.0841; found 289.0841.

*(E)-1-(4-Bromophenyl)-3-(4-dimethylamino)phenylprop-2-en-1-one* (**2k**) [46]. Pale yellow powder, 60% yield, m.p. = 140–142 °C; ^1^H-NMR (CDCl_3_, 300 MHz): δ = 3.07 (s, 6H, N(CH_3_)_2_), 6.72 (d, 2H, *J* = 8.7 Hz, Ar-), 7.30 (d, 1H, *J* = 15.4 Hz, CH=), 7.54 (d, 2H, *J* = 8.7 Hz, Ar-), 7.62 (d, 2H, *J* = 8.7 Hz, Ar-), 7.74 (d, 1H, *J* = 15.4 Hz, CH=), 7.89 (d, 2H, J = 8.7 Hz, Ar-) ppm; ^13^C-NMR (CDCl_3_, 75 MHz): δ = 40.1, 111.8, 116.2, 122.4, 127.1, 129.9, 130.6, 131.7, 137.9, 146.4, 152.2, 189.4 ppm; HRMS (ESI^+^): *m*/*z* calcd for C_17_H_16_BrNO [M + Na]^+^ 352.0313; found 352.0314.

*(E)-1-(4-Bromophenyl)-3-(4-methoxyphenyl)prop-2-en-1-one* (**2l**) [46]. Pale cream powder, 59% yield, m.p. = 148–150 °C; ^1^H-NMR (CDCl_3_, 300 MHz): δ = 3.88 (s, 3H, OCH_3_), 6.97 (d, 2H, *J* = 8.8 Hz, Ar-), 7.38 (d, 1H, *J* = 15.6 Hz, CH=), 7.63 (d, 2H, *J* = 8.8 Hz, Ar-), 7.66 (d, 2H, *J* = 8.6 Hz, Ar-), 7.82 (d, 1H, *J* = 15.6 Hz, CH=), 7.90 (d, 2H, *J* = 8.6 Hz, Ar-) ppm; ^13^C-NMR (CDCl_3_, 75 MHz): δ = 55.7, 114.7, 119.3, 127.6, 127.8, 130.2, 130.6, 132.1, 137.4, 145.5, 162.1, 189.6 ppm; HRMS (ESI^+^): *m*/*z* calcd for C_16_H_13_BrO_2_ [M + Na]^+^ 338.9997; found 338.9997.

*(E)-3-(4-Methoxyphenyl)-1-(4-nitrophenyl)prop-2-en-1-one* (**2m**) [43]. Yellow powder, 65% yield, m.p. = 183–185 °C; ^1^H-NMR (CDCl_3_, 300 MHz): δ = 3.89 (s, 3H, OCH_3_), 6.97 (d, 2H, *J* = 8.7 Hz, Ar-), 7.37 (d, 1H, *J* = 15.6 Hz, CH=), 7.64 (d, 2H, *J* = 8.7 Hz, Ar-), 7.84 (d, 1H, *J* = 15.6 Hz, CH=), 8.14 (d, 2H, *J* = 8.9 Hz, Ar-), 8.36 (d, 2H, *J* = 8.9 Hz, Ar-) ppm; ^13^C-NMR (CDCl_3_, 75 MHz): δ = 55.5, 114.6, 118.9, 123.9, 127.0, 129.3, 130.5, 143.5, 146.7, 149.9, 162.3, 189.1 ppm. HRMS (ESI^+^): *m*/*z* calcd for C_16_H_13_NO_4_ [M + Na]^+^ 306.0742; found 306.0742.

*Methyl (E)-4-(3-(4-bromophenyl)acryloyl)benzoate* (**2n**). Pale yellow powder, 62% yield, m.p. = 212–214 °C; ^1^H-NMR (CDCl_3_, 300 MHz): δ = 3.96 (s, 3H, OCH_3_), 7.40–7.51 (m, 5H, CH=, Ar-), 7.68 (d, 1H, *J* = 15.7 Hz, CH=), 7.97 (d, 2H, *J* = 8.5 Hz, Ar-), 8.10 (d, 2H, *J* = 8.5 Hz, Ar-) ppm; ^13^C-NMR (CDCl_3_, 75 MHz): δ = 52.5, 122.3, 125.2, 128.1, 128.4, 129.9, 132.3, 133.5, 133.7, 141.4, 144.3, 189.8 ppm. HRMS (ESI^+^): *m*/*z* calcd for C_17_H_13_BrO_3_ [M + Na]^+^ 366.9946; found 366.9946.

*Methyl (E)-4-(3-(4-dimethylamino)phenyl)acryloyl)-benzoate* (**2o**). Orange powder, 58% yield, m.p. = 164–166 °C; ^1^H-NMR (CDCl_3_, 300 MHz): δ = 3.10 (s, 3H, N(CH_3_)_2_), 3.96 (s, 3H, OCH_3_), 6.71 (d, 2H, *J* = 8.8 Hz, Ar-), 7.30 (d, 1H, *J* = 15.5 Hz, CH=), 7.55 (d, 2H, *J* = 8.8 Hz, Ar-), 7.79 (d, 1H, *J* = 15.5 Hz, CH=), 8.02 (d, 2H, *J* = 8.6 Hz, Ar-), 8.14 (d, 2H, *J* = 8.6 Hz, Ar-) ppm; ^13^C-NMR (CDCl_3_, 75 MHz): δ = 40.0, 52.3, 111.7, 116.4, 122.2, 128.1, 129.6, 130.6, 132.8, 142.6, 146.8, 152.2, 166.4, 190.0 ppm. HRMS (ESI^+^): *m*/*z* calcd for C_19_H_19_NO_3_ [M + Na]^+^ 322.1263; found 322.1262. 

*Methyl (E)-4-(3-methoxyphenyl)acryloyl)-benzoate* (**2p**). Pale yellow powder, 57% yield, m.p. = 144–146 °C; ^1^H-NMR (CDCl_3_, 300 MHz): δ = 3.83 (s, 3H, OCH_3_), 3.90 (s, 3H, OCH_3_), 6.94 (d, 2H, *J* = 8.7 Hz, Ar-), 7.38 (d, 1H, *J* = 15.6 Hz, CH=), 7.61 (d, 2H, *J* = 8.7 Hz, Ar-), 7.79 (d, 1H, *J* = 15.6 Hz, CH=), 8.03 (d, 2H, *J* = 8.4 Hz, Ar-), 8.15 (d, 2H, *J* = 8.4 Hz, Ar-) ppm; ^13^C-NMR (CDCl_3_, 75 MHz): δ = 52.3, 55.3, 114.3, 119.4, 127.2, 128.1, 129.6, 130.3, 133.1, 141.8, 145.5, 161.8, 166.2, 190.0 ppm; HRMS (ESI^+^): *m*/*z* calcd for C_18_H_16_O_4_ [M + Na]^+^ 319.0946; found 319.0945.

*(Z)-5-(Benzo[d][1,3]dioxol-5-ylmethylene)-2-thioxo-imidazolidin-4-one* (**3a**) [47]. Yellow powder, 75% yield, m.p. ≥ 260 °C; ^1^H-NMR (DMSO-*d*_6_, 300 MHz): δ = 6.09 (s, 2H, OCH_2_O), 6.43 (s, 1H, CH=), 6.97 (d, 1H, *J* = 8.1 Hz, Ar-), 7.27 (d, 1H, *J* = 8.1 Hz, Ar-), 7.44 (s, 1H, Ar-), 12.08 (br s, 1H, NH), 12.31 (br s, 1H, NH) ppm; ^13^C-NMR (DMSO-*d*_6_, 75 MHz): δ = 102.1, 109.2, 109.7, 112.6, 126.5, 126.7, 126.9, 148.4, 148.9, 166.2, 179.1 ppm; HRMS (ESI^+^): *m*/*z* calcd for C_11_H_8_N_2_O_3_S [M + Na]^+^ 271.0153; found 271.0152.

*(Z)-5-(4-Methoxybenzylidene)-2-thioxoimidazolidin-4-one* (**3b**) [48]. Yellow powder, 67% yield, m.p. = 211–213 °C; ^1^H-NMR (DMSO-*d*_6_, 300 MHz): δ = 3.81 (s, 3H, OCH_3_), 6.47 (s, 1H, CH=), 6.98 (d, 2H, *J* = 8.8 Hz, Ar-), 7.74 (d, 2H, *J* = 8.8 Hz), 12.07 (br s, 1H, NH), 12.30 (s, 1H, NH) ppm; ^13^C-NMR (DMSO-*d*_6_, 75 MHz): δ = 55.4, 113.9, 114.4, 124.0, 124.7, 127.4, 128.3, 132.3, 160.4, 163.9, 177.9 ppm; HRMS (ESI^+^): *m*/*z* calcd for C_11_H_10_N_2_O_2_S [M + Na]^+^ 257.0361; found 257.0361.

*(Z)-5-(4-Methoxybenzylidene)imidazolidine-2*,*4-dione* (**3c**) [38]. Yellow powder, 49% yield, m.p. = 202–204 °C; ^1^H-NMR (DMSO-*d*_6_, 300 MHz): δ = 3.79 (s, 3H, OCH_3_), 6.38 (s, 1H, CH=), 6.95 (d, 2H, *J* = 8.8 Hz, Ar-), 7.58 (d, 2H, *J* = 8.8 Hz, Ar-), 10.42 (br s, 1H, NH), 11.16 (br s, 1H, NH) ppm; ^13^C-NMR (DMSO-*d*_6_, 75 MHz): δ = 55.2, 108.6, 114.1, 125.4, 131.0, 155.6, 159.4, 165.5, 173.9 ppm; HRMS (ESI^+^): *m*/*z* calcd for C_11_H_10_N_2_O_3_ [M + Na]^+^ 241.0589; found 241.0591.

*(Z)-5-(4-Methoxybenzylidene)-2-thioxothiazolidin-4-one* (**3d**) [38]. Yellow powder, 90% yield, m.p. = 263–265 °C; ^1^H-NMR (DMSO-*d*_6_, 300 MHz): δ = 3.84 (s, 3H, OCH_3_), 7.11 (d, 2H, *J* = 8.7 Hz, Ar-), 7.57 (d, 2H, *J* = 8.7 Hz, Ar-), 7.60 (s, 1H, CH=), 13.75 (br s, 1H, NH) ppm; ^13^C-NMR (DMSO-*d*_6_, 75 MHz): δ = 55.4, 114.9, 124.9, 125.8, 129.7, 132.2, 160.8, 172.9, 197.2 ppm; HRMS (ESI^+^): *m*/*z* calcd for C_11_H_9_NO_2_S_2_ [M + Na]^+^ 273.9972; found 273.9972.

*(Z)-5-(Benzo[d][1,3]dioxol-5-ylmethylene)-2-thioxo-thiazolidin-4-one* (**3e**) [49]. Yellow powder, 95% yield, m.p. = 248–250 °C; ^1^H-NMR (DMSO-*d*_6_, 300 MHz): δ = 6.13 (s, 2H, OCH_2_O), 7.11 (m, 3H, Ar-), 7.54 (s, 1H, CH=), 13.74 (br s, 1H, NH) ppm; ^13^C-NMR (DMSO-*d*_6_, 75 MHz): δ = 102.5, 109.6, 121.4, 126.3, 127.6, 132.4, 148.6, 149.7, 167.8, 195.3 ppm; HRMS (ESI^+^): *m*/*z* calcd for C_11_H_7_NO_3_S_2_ [M + Na]^+^ 287.9765; found 287.9765.

*(Z)-5-(4-Chlorobenzylidene)-2-thioxothiazolidin-4-one* (**3f**) [50]. Pale orange powder, 91% yield, m.p. = 230–232 °C; ^1^H-NMR (DMSO-*d*_6_, 300 MHz): δ = 7.60–7.64 (m, 5H, CH=, Ar-), 13.89 (br s, 1H, NH) ppm; ^13^C-NMR (DMSO-*d*_6_, 75 MHz): δ = 126.2, 129.5, 130.1, 131.8, 132.0, 135.3, 169.3, 195.4 ppm; HRMS (ESI^+^): *m*/*z* calcd for C_10_H_6_ClNOS_2_ [M + Na]^+^ 277.9477; found 277.9476.

*(Z)-5-(4-(Dimethylamino)benzylidene)-2-thioxo-thiazolidin-4-one* (**3g**) [51]. Orange powder, 90% yield, m.p. = 270–272 °C; ^1^H-NMR (DMSO-*d*_6_, 300 MHz): δ = 3.04 (s, 6H, N(CH_3_)_2_), 6.83 (d, 2H, *J* = 8.7 Hz, Ar-), 7.43 (d, 2H, *J* = 8.7 Hz, Ar-), 7.54 (s, 1H, CH=), 13.59 (br s, 1H, NH) ppm; ^13^C-NMR (DMSO-*d*_6_, 75 MHz): δ = 40.3, 112.5, 117.7, 120.1, 133.3, 133.7, 152.1, 169.8, 195.4 ppm; HRMS (ESI^+^): *m*/*z* calcd for C_12_H_12_N_2_OS_2_ [M + Na]^+^ 287.0289; found 287.0287.

*(Z)-5-(4-Nitrobenzylidene)-2-thioxothiazolidin-4-one* (**3h**) [52]. Yellow powder, 93% yield, m.p. = 254–256 °C; ^1^H-NMR (DMSO-*d*_6_, 300 MHz): δ = 7.75 (s, 1H, CH=), 7.87 (d, 2H, *J* = 8.6 Hz, Ar-), 8.35 (d, 2H, *J* = 8.6 Hz, Ar-), 14.03 (br s, 1H, NH) ppm; ^13^C-NMR (DMSO-*d*_6_, 75 MHz): δ = 124.7, 128.7, 130.3, 131.7, 139.6, 147.9, 169.6, 196.7 ppm; HRMS (ESI^+^): *m*/*z* calcd for C_10_H_6_N_2_O_3_S_2_ [M + Na]^+^ 288.9718; found 288.9719.

*(Z)-5-(4-Hydroxy-3-methoxybenzylidene)-2-thioxothiazolidin-4-one* (**3i**) [38]. Yellow powder, 79% yield, m.p. = 228–230 °C; ^1^H-NMR (DMSO-*d*_6_, 300 MHz): δ = 3.85 (s, 3H, OCH_3_), 6.94–7.17 (m, 3H, Ar-), 7.60 (s, 1H, CH=), 10.13 (br s, 1H, OH), 13.75 (br s, 1H, NH) ppm; ^13^C-NMR (DMSO-*d*_6_, 75 MHz): δ = 56.1, 114.8, 116.8, 121.6, 124.8, 125.5, 133.2, 148.6, 150.4, 169.9, 195.9 ppm; HRMS (ESI^+^): *m*/*z* calcd for C_11_H_9_NO_3_S_2_ [M + Na]^+^ 299.9922; found 299.9923.

*(Z)-5-(4-Methoxybenzylidene)thiazolidine-2*,*4-dione* (**3j**) [53]. Yellow powder, 44% yield, m.p. = 214–216 °C; ^1^H-NMR (DMSO-*d*_6_, 300 MHz): δ = 3.22 (s, 3H, OCH_3_), 7.10 (d, 2H, *J* = 8.7 Hz, Ar-), 7.56 (d, 2H, *J* = 8.7 Hz, Ar-), 7.75 (s, 1H, CH=), 12.51 (br s, 1H, NH) ppm; ^13^C-NMR (DMSO-*d*_6_, 75 MHz): δ = 55.5, 114.9, 125.6, 131.6, 132.0, 160.9, 168.1, 168.3 ppm; HRMS (ESI^+^): *m*/*z* calcd for C_11_H_9_NO_3_S [M + Na]^+^ 258.0211; found 258.0212.

*(Z)-5-(4-Benzilydene-4-boronic acid)-2-thioxo-imidazolidin-4-one* (**4**). Pale Brown powder, 65% yield, m.p. = 242–244 °C; ^1^H-NMR (DMSO-*d*_6_, 300 MHz): δ = 7.54 (d, 2H, *J* = 8.1 Hz, Ar-), 7.62 (s, 1H, CH=), 7.91 (d, 2H, *J* = 8.1 Hz, Ar-), 13.85 (s, 1H, NH) ppm; ^13^C-NMR (DMSO-*d*_6_, 75 MHz): δ = 126.3, 129.8, 132.1, 134.7, 135.4, 169.8, 196.2 ppm (Carbon atoms in α to boron are often non visible in ^13^C-NMR); ^11^B NMR (96 MHz, DMSO-*d*_6_): 28.30; HRMS (ESI^+^): *m*/*z* calcd for C_10_H_8_BNO_3_S_2_ [M + Na]^+^ 287.9936; found 287.9939.

*(E)-1-(4-Methoxyphenyl)-3-phenylprop-2-en-1-one* (**5**) [40]. Pale yellow powder, 50% yield, m.p. = 106–108 °C; ^1^H-NMR (CDCl_3_, 300 MHz): δ = 3.90 (s, 3H, OCH_3_), 6.98 (d, 2H, *J* = 8.8 Hz, Ar-), 7.40–7.45 (m, 3H, Ar-), 7.55 (d, 1H, *J* = 15.7 Hz, CH=), 7.62–7.64 (m, 2H, Ar-), 7.80 (d, 1H, *J* = 15.7 Hz, CH=), 8.03 (d, 2H, *J* = 8.8 Hz, Ar-) ppm; ^13^C-NMR (CDCl_3_, 75 MHz): δ = 55.5, 113.8, 121.9, 128.2, 128.3, 128.9, 130.3, 130.8, 144.0, 163.4, 188.8 ppm. HRMS (ESI^+^): *m*/*z* calcd for C_16_H_14_O_2_ [M + Na]^+^ 261.0891; found 261.0892.

## 4. Conclusions

We described here an environmentally friendly protocol under microwave irradiation conditions for the preparation of several condensation products. The reaction time is short and non-toxic and cheap boric acid is employed as catalyst. These conditions can be considered as an alternative method to those traditionally described by their compatibility to base-sensitive functional groups. Our preliminary attempts to develop an auto-catalyzed reaction with a substrate bearing a boronic acid function have partly failed. Work is ongoing in our laboratory.

## Supplementary Materials

Supplementary materials can be accessed at: http://www.mdpi.com/1420-3049/20/06/11617/s1.
