# Supplementary material for: Microwave-Assisted Condensation Reactions of Acetophenone Derivatives and Activated Methylene Compounds with Aldehydes Catalyzed by Boric Acid under Solvent-Free Conditions
Source: Molecules. 2015 Jun 23;20(6):11617–31. doi: 10.3390/molecules200611617 (PMC6272727; doi:10.3390/molecules200611617)
Supplement: Supplementary file 1 [file molecules-20-11617-s001.pdf]

Supplementary Materials

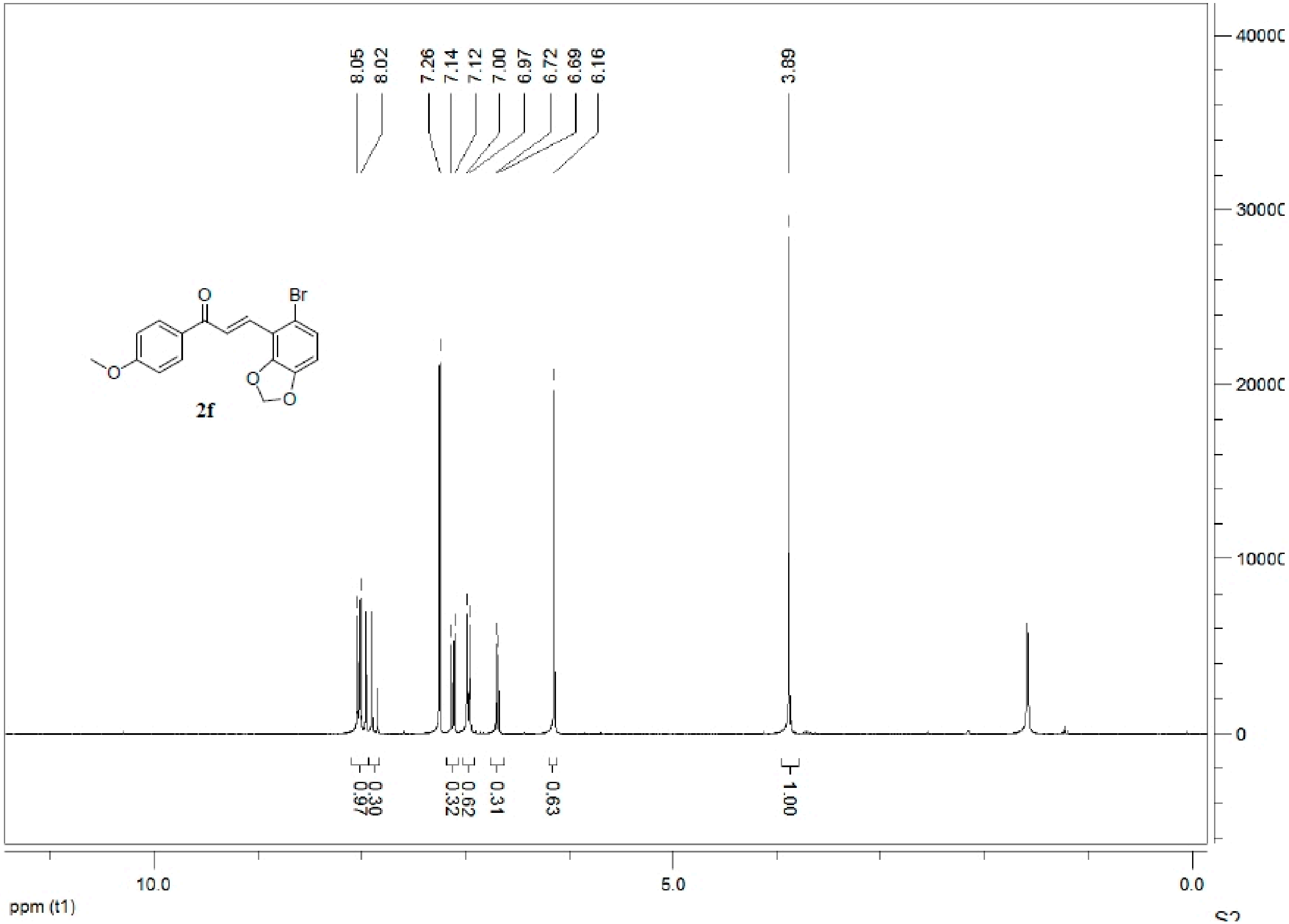

Figure S1. ¹H-NMR of **2f**.

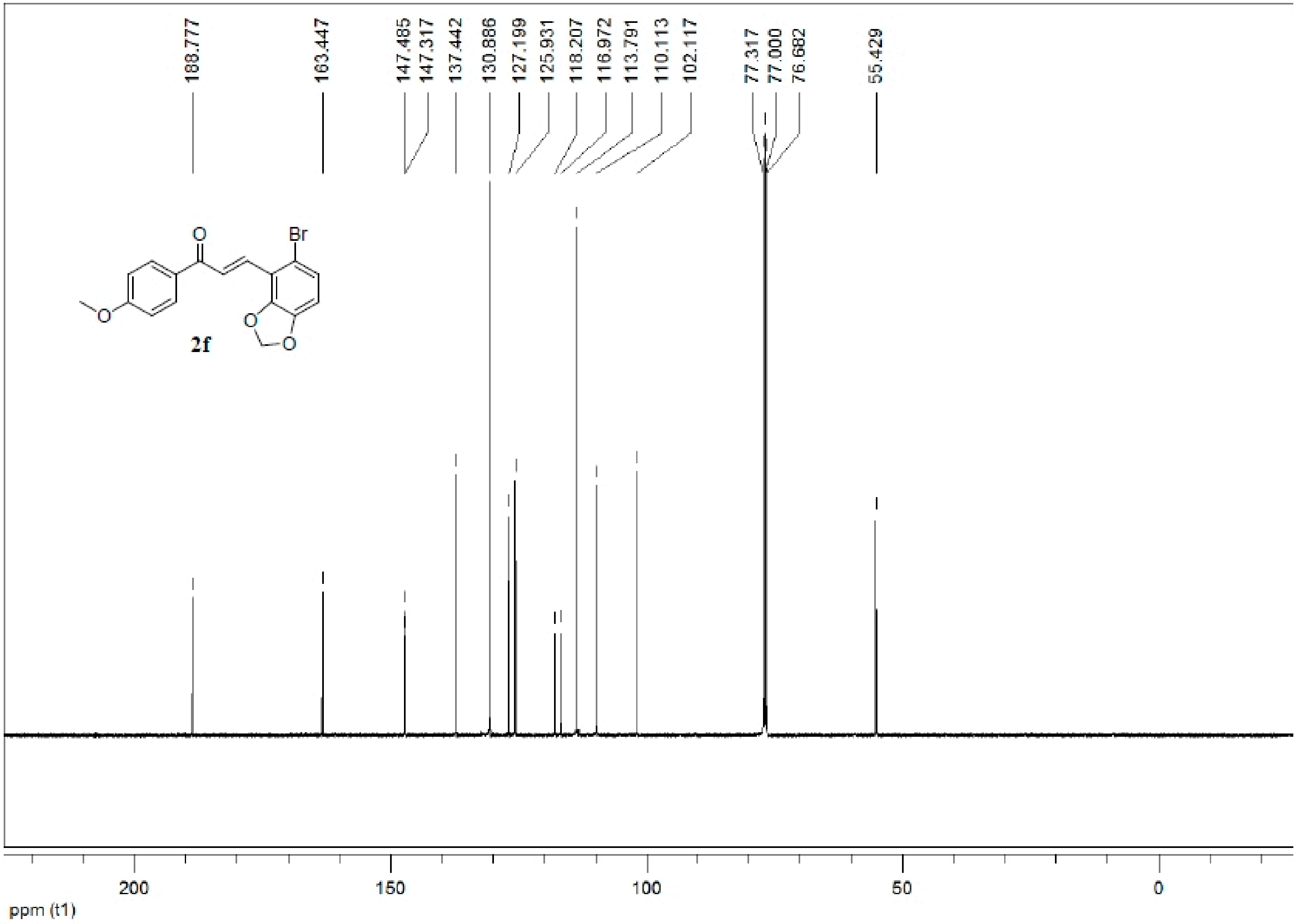

Figure S2. ^{13}C-NMR of **2f**.

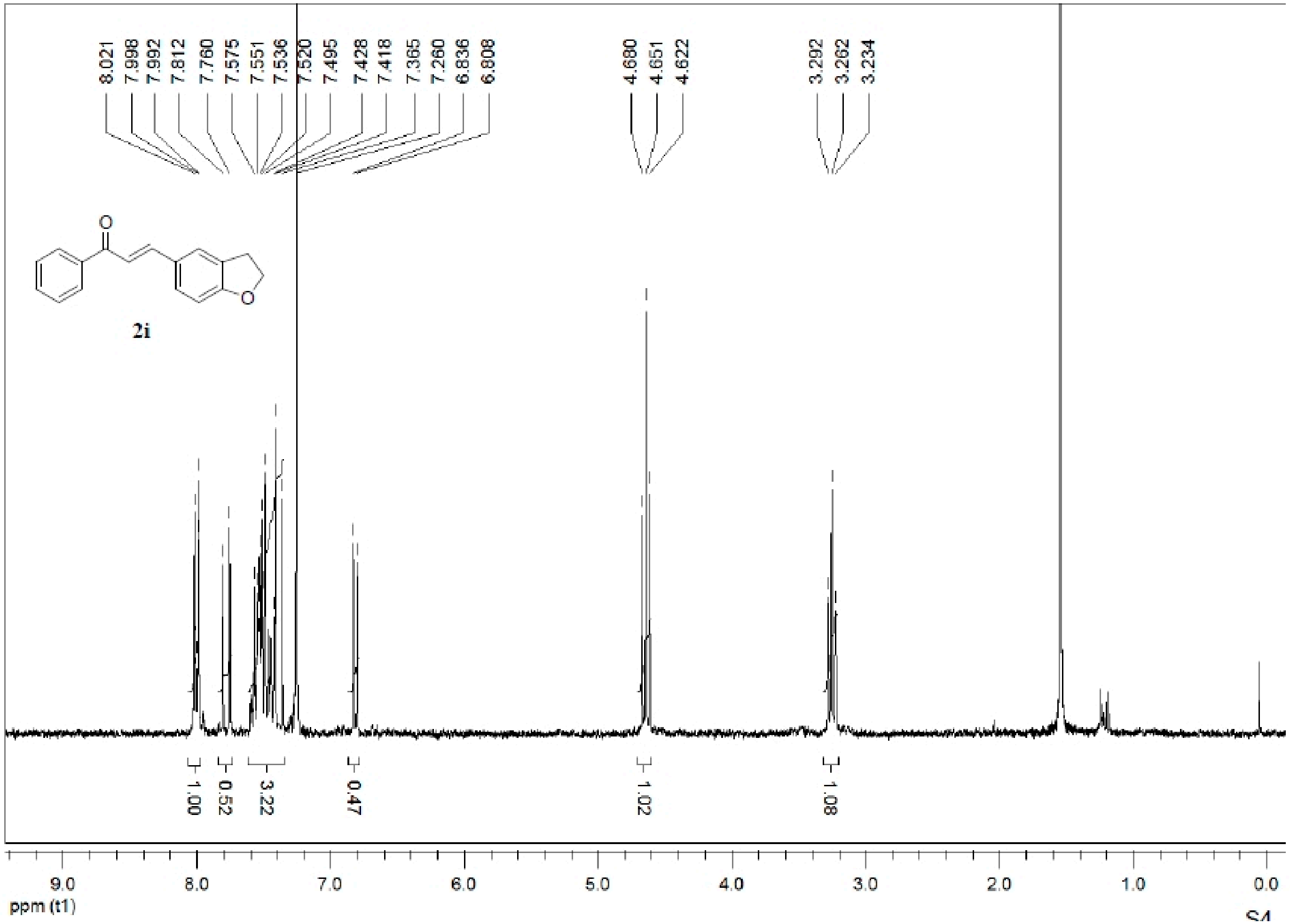

Figure S3. ^{1}H-NMR of **2i**.

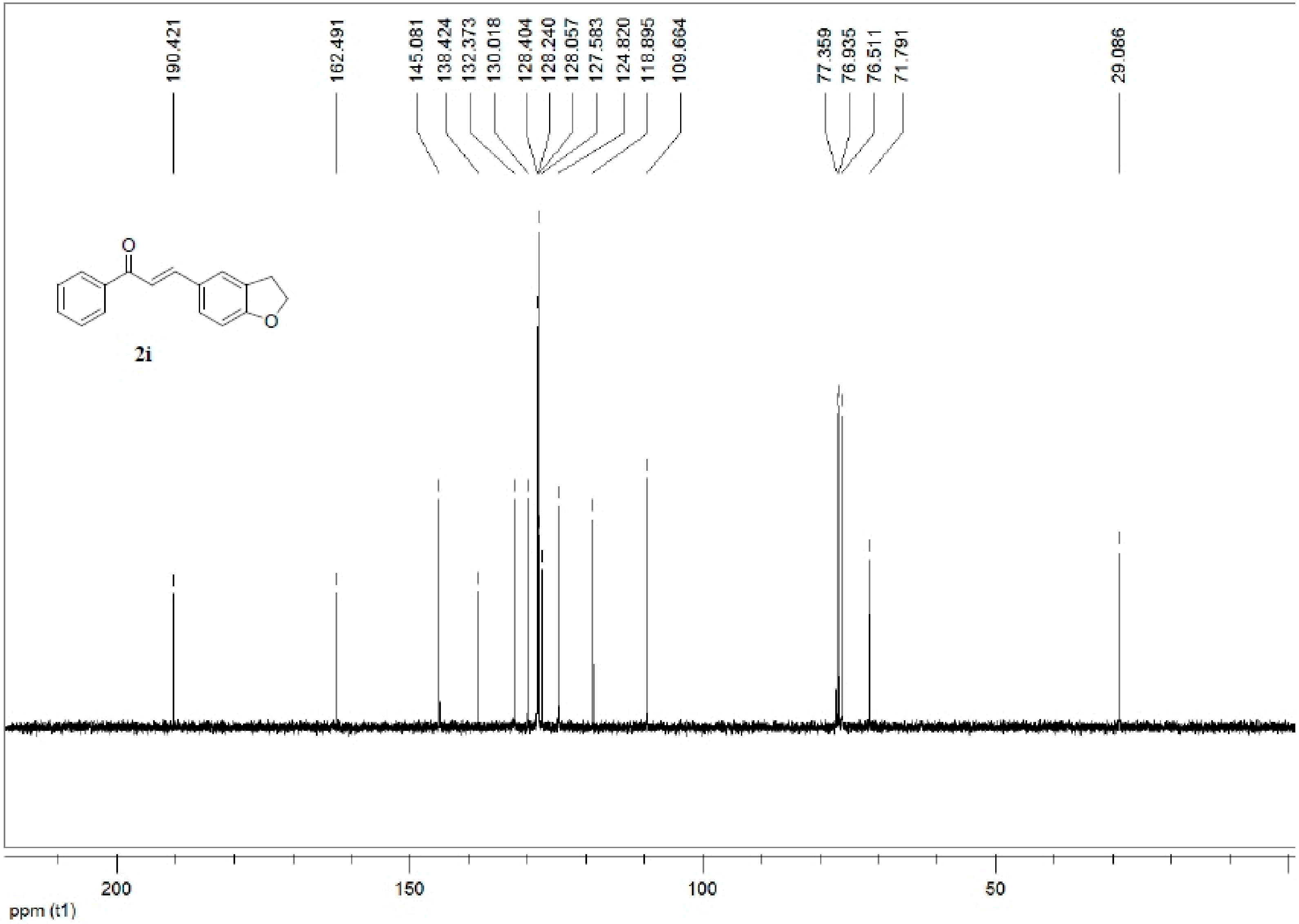

Figure S4. ^{13}C-NMR of **2i**.

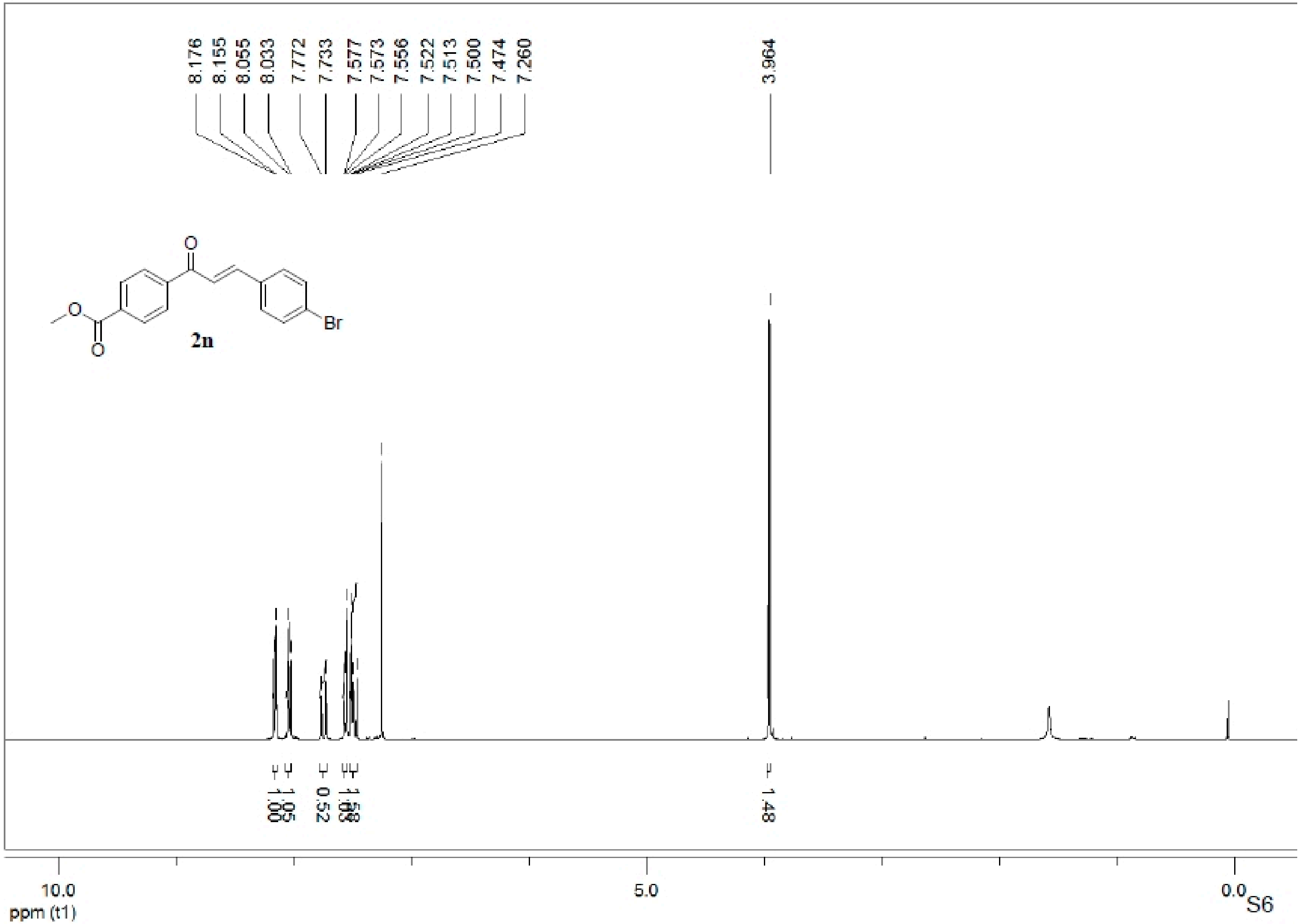

Figure S5. ^{1}H-NMR of **2n**.

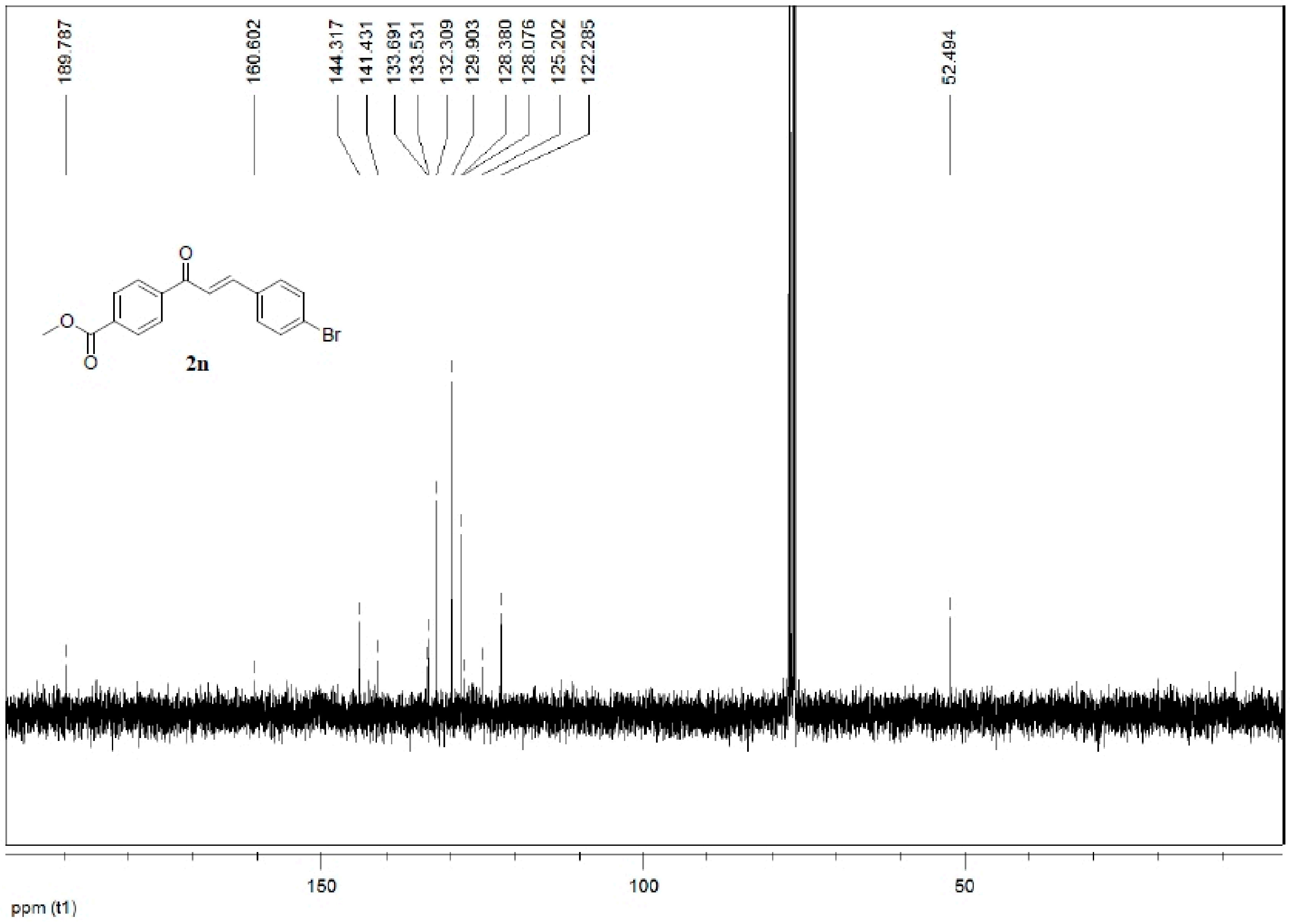

Figure S6. ^{13}C-NMR of **2n**.

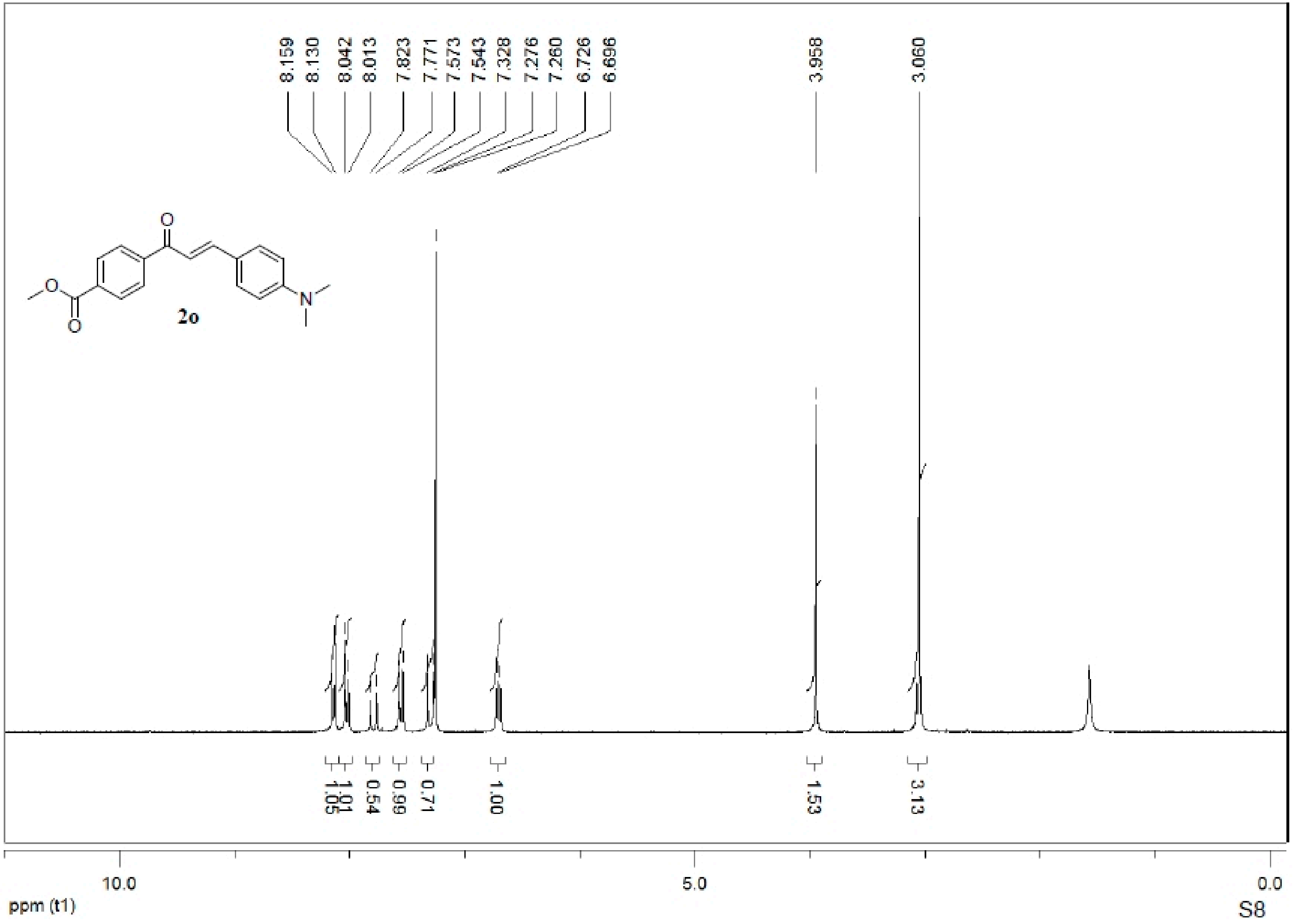

Figure S7. ^{1}H-NMR of **2o**.

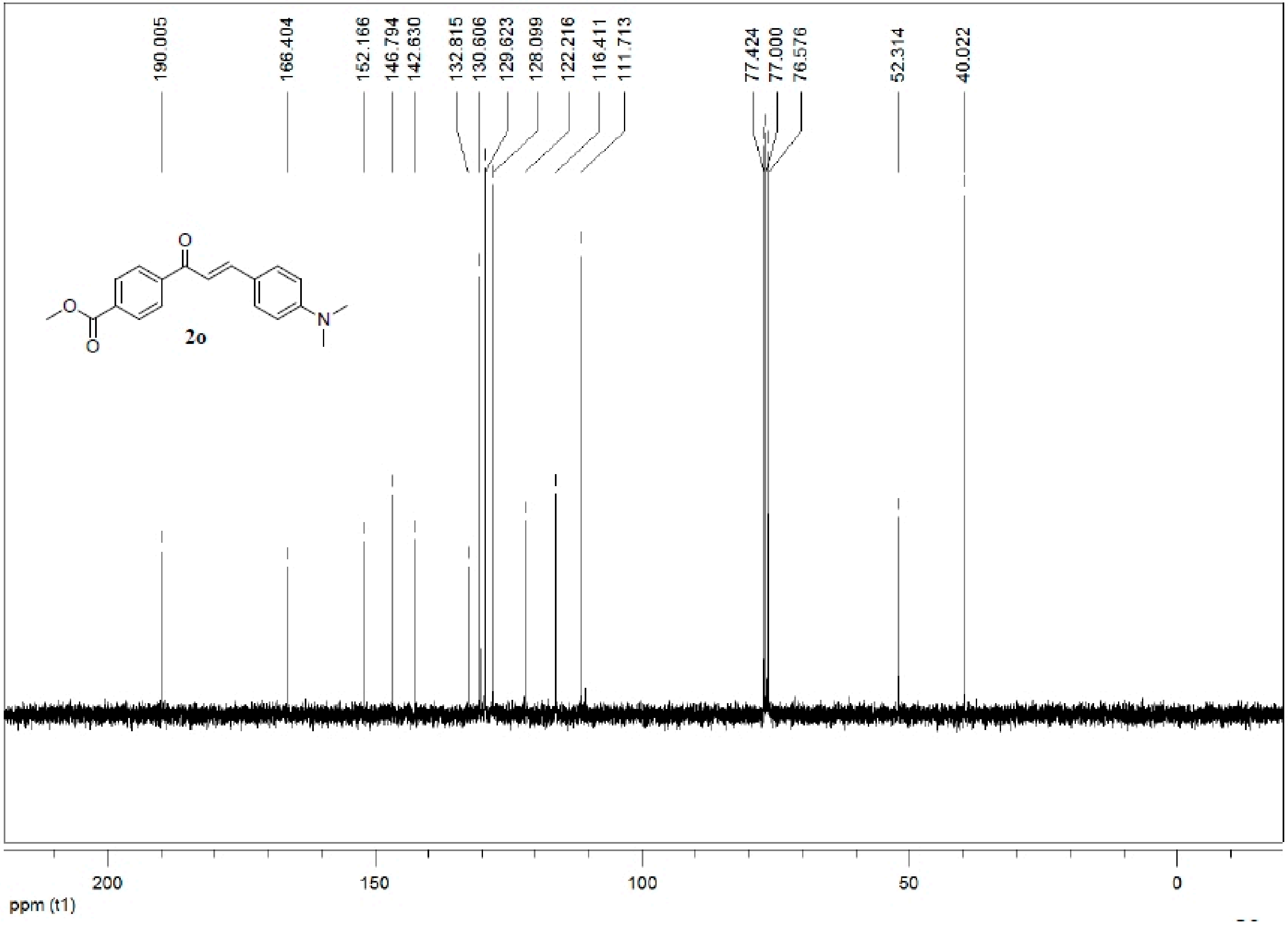

Figure S8. 13C-NMR of **2o**.

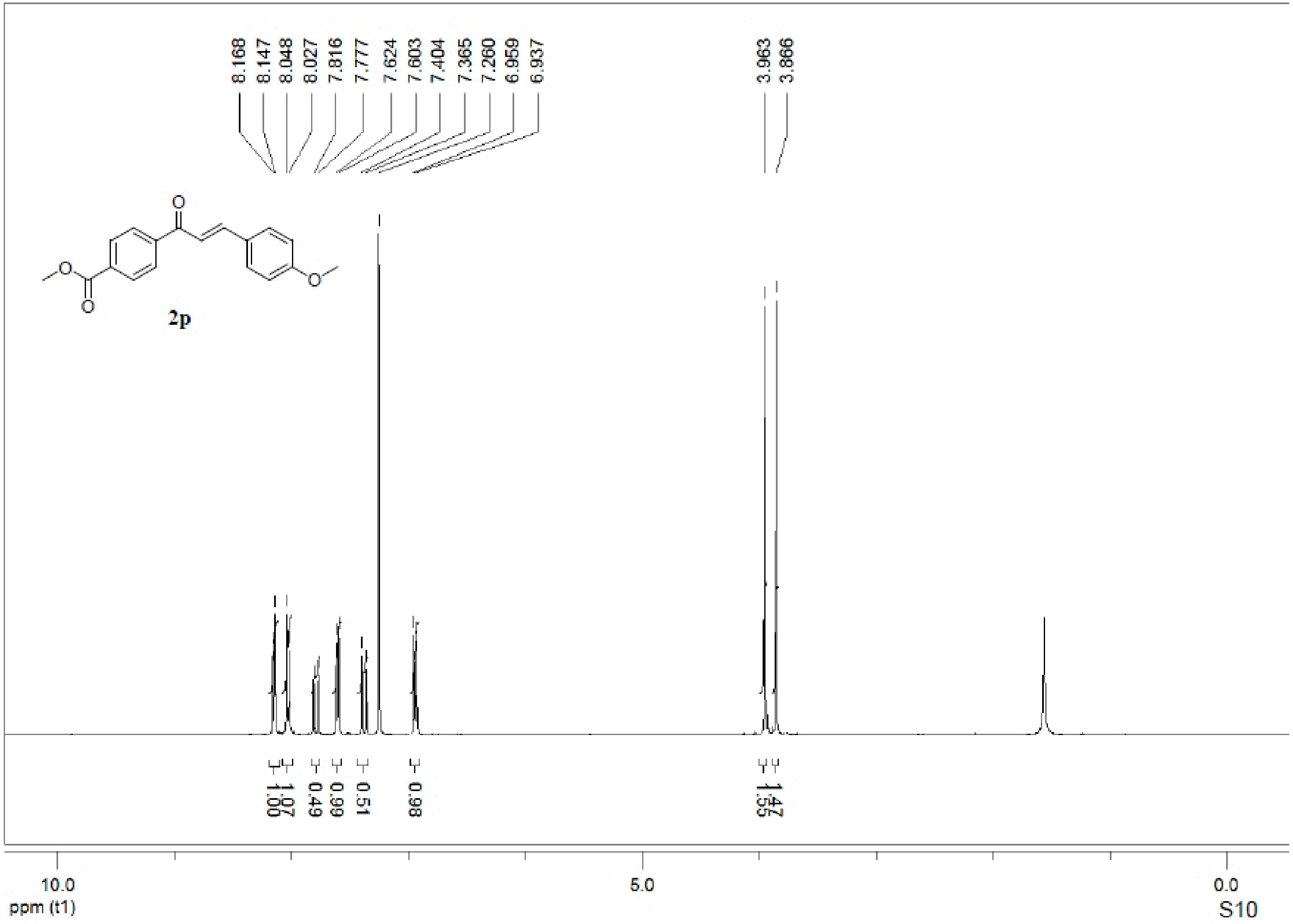

Figure S9. ^{1}H-NMR of **2p**.

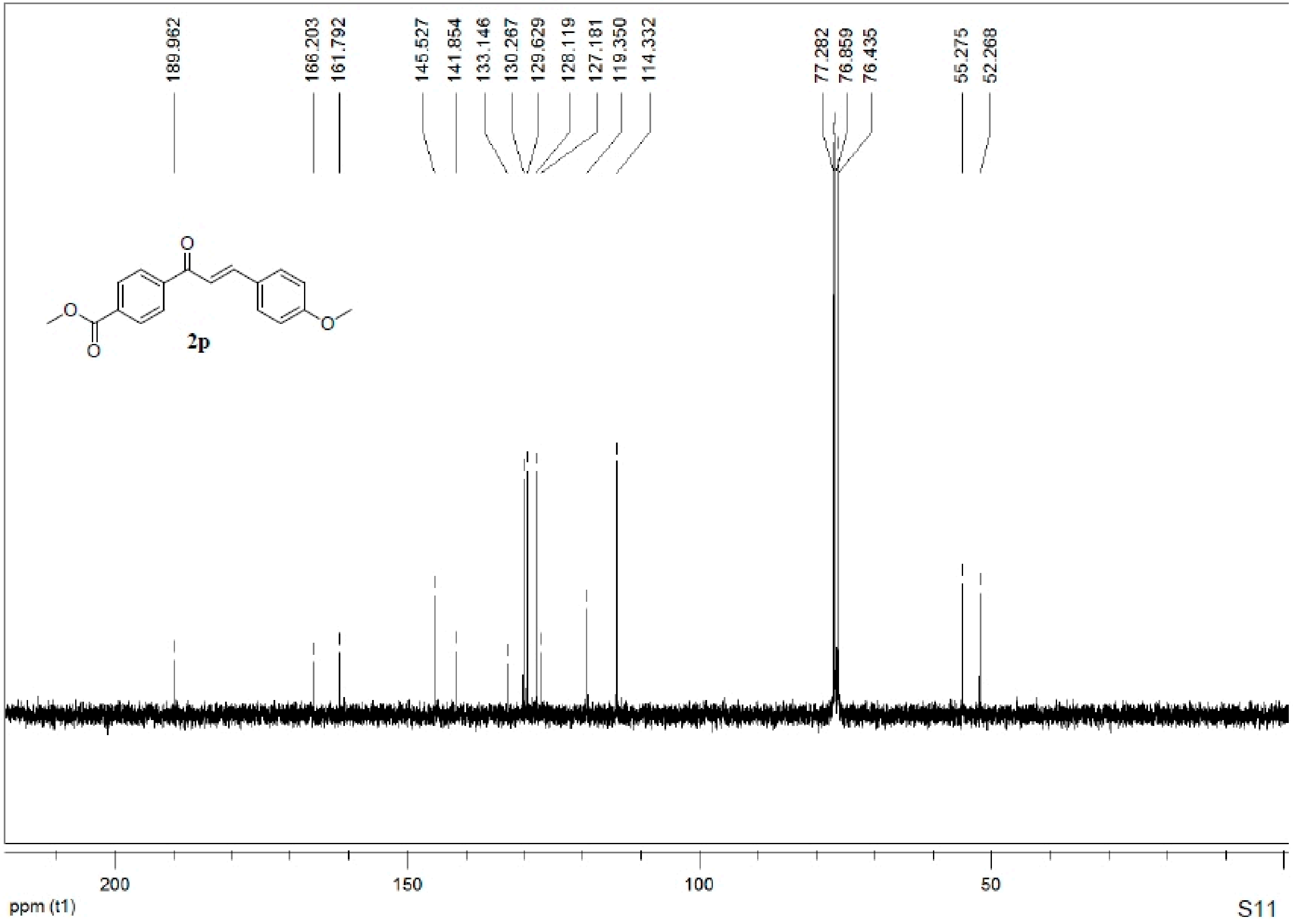

Figure S10. ^{13}C-NMR of **2p**.

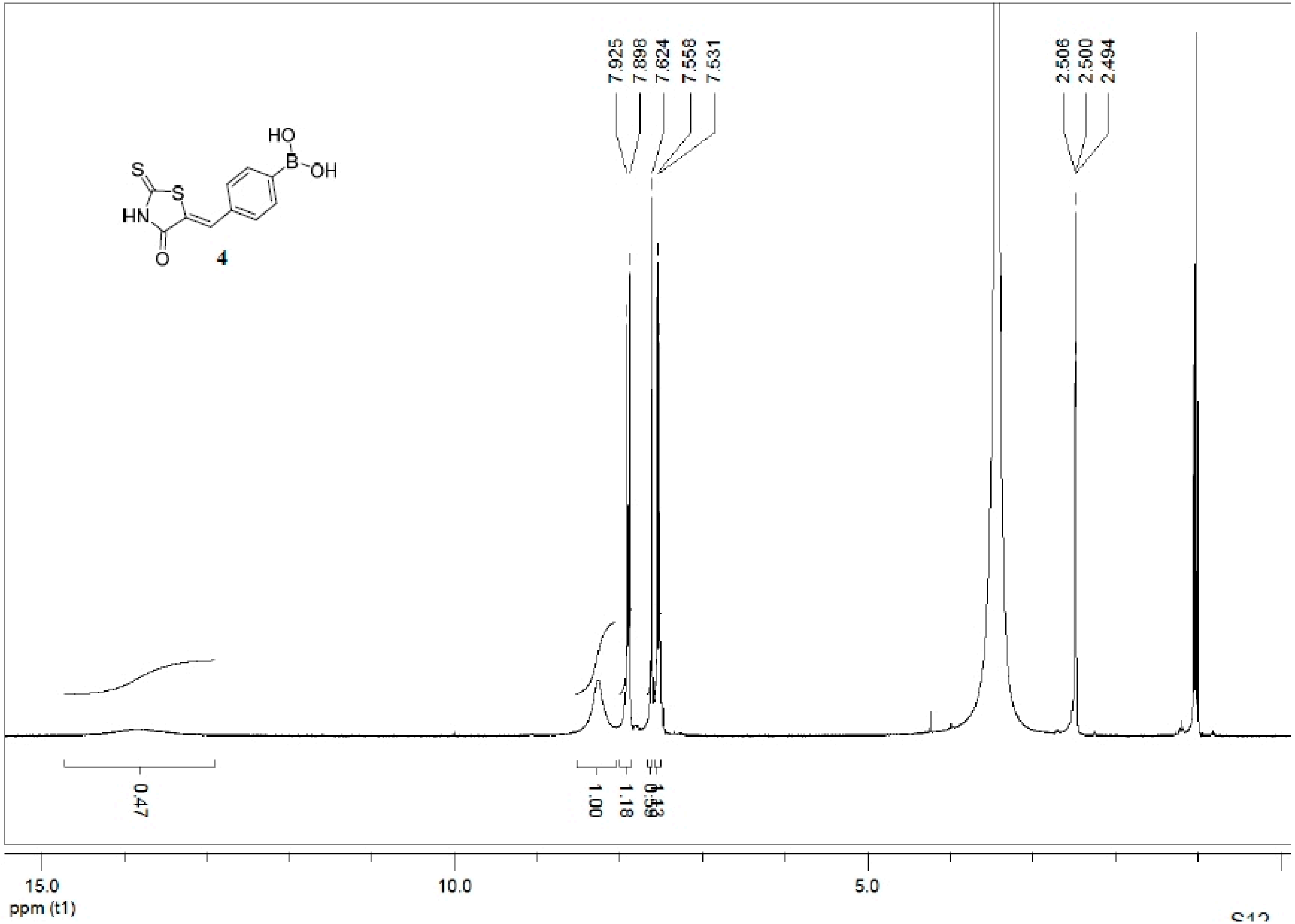

Figure S11. ^{1}H-NMR of **4**.

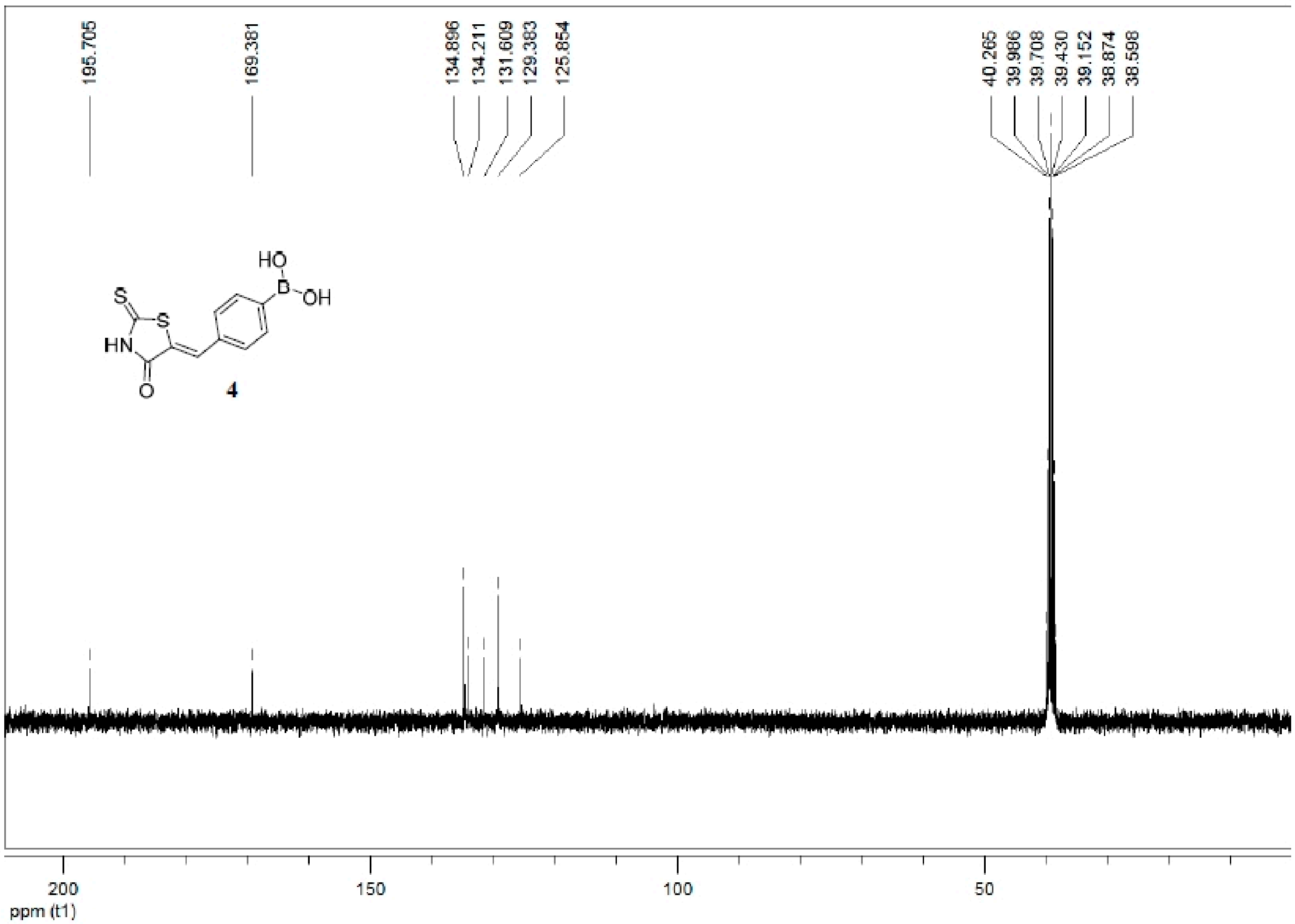

Figure S12. ^{13}C-NMR of **4.**

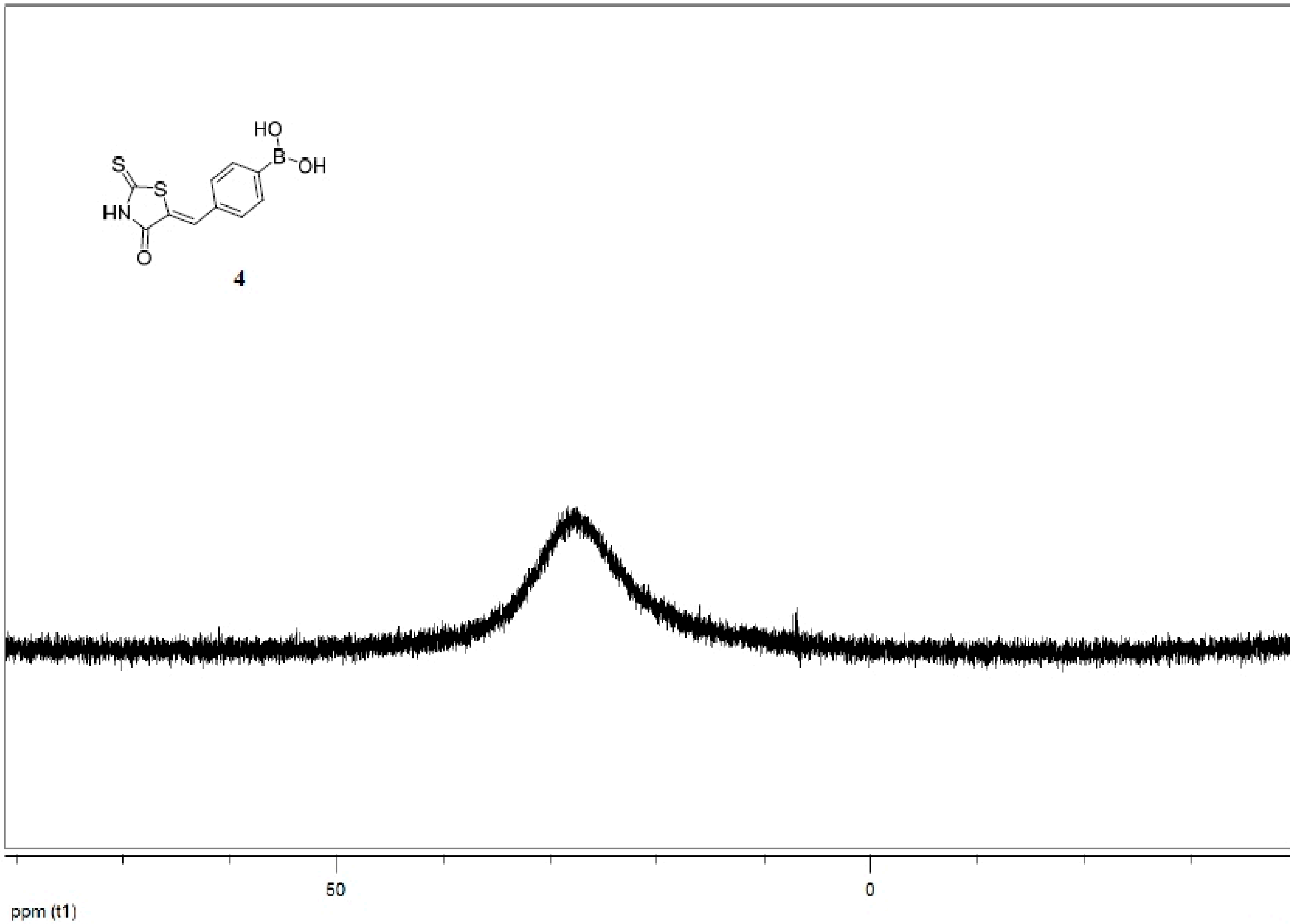

Figure S13. ^{11}B-NMR of **4.**
